# Predictive value of neutrophil to lymphocyte ratio and platelet to lymphocyte ratio for acute deep vein thrombosis after total joint arthroplasty: a retrospective study

**DOI:** 10.1186/s13018-018-0745-x

**Published:** 2018-02-27

**Authors:** Chen Yao, Zhe Zhang, Yao Yao, Xingquan Xu, Qing Jiang, Dongquan Shi

**Affiliations:** 1 Department of Sports Medicine and Adult Reconstructive Surgery, Nanjing Drum Tower Hospital affiliated with the Medical School of Nanjing University, Nanjing, Jiangsu China; 2 Laboratory for Bone and Joint Disease, Model Animal Research Center, Nanjing University, Nanjing, Jiangsu China

**Keywords:** Neutrophil to lymphocyte ratio, Platelet to lymphocyte ratio, Deep vein thrombosis, Total joint arthroplasty

## Abstract

**Background:**

Deep vein thrombosis (DVT) is a common and severe complication of total joint arthroplasty (TJA). Inflammation has been proved to play a role in DVT. The neutrophil to lymphocyte ratio (NLR) and the platelet to lymphocyte ratio (PLR) are biomarkers for systemic inflammation. The aim of the study is to investigate the predictive value of NLR and PLR for acute TJA-induced DVT.

**Method:**

A total of 773 patients who underwent primary TJA in our hospital were included in this retrospective study. Venography was performed routinely after the surgery to define acute DVT. NLR and PLR before and after operation were calculated according to the blood routine test. Multiple logistic regression analyses and ROC curve analyses were performed to assess the association of NLR and PLR with TJA-induced DVT.

**Results:**

One hundred twenty out of 773 patients (15.5%) were diagnosed with DVT by venography. In patients with DVT, preoperative NLR (*P* = 0.030) and postoperative NLR (*P* = 0.015) were significantly higher but postoperative PLR (*P* = 0.002) was significantly lower. Multiple logistic regression analyses indicated that age (OR = 1.05, *P* < 0.005), gender (OR = 0.47, *P* = 0.005), BMI (OR = 1.06, *P* < 0.014), preoperative NLR (OR = 1.11, *P* < 0.035), postoperative NLR (OR = 1.20, *P* < 0.001), and PLR (OR = 0.99, *P* < 0.001) were independently associated with DVT. However, the ROC curve analysis demonstrated the specificity and sensitivity of NLR or PLR in predicting DVT were low.

**Conclusion:**

Although the present study demonstrated significant association of perioperative NLR or PLR with acute TJA-induced DVT, NLR or PLR cannot predict TJA-induced DVT accurately.

## Background

Total joint arthroplasty (TJA), including total knee arthroplasty (TKA) and total hip arhroplasty (THA), has been performed widely for end-stage diseases of the knee and hip. Deep vein thrombosis (DVT) is a common and severe complication of TJA, especially in hospitalized patients [1, 2]. DVT can lead to pulmonary embolism (PE), which may occur rapidly and cause immediate death. Although the mechanical and pharmacological prophylaxis significantly reduced the incidence of postsurgical DVT, it remains a major cause of postoperative morbidity and mortality [3]. Lack of subject symptoms and clinical signs make diagnosis of DVT complicated. Venography and ultrasound are commonly used diagnostic techniques for DVT, which are inconvenient, costly, or invasive. Many studies have focused on the serology, trying to find valuable biomarkers of DVT such as D-dimer, P-selection, Factor VII, and so on [4–6].

Increasing evidence suggests that inflammation is involved not only in the pathophysiology of arterial thrombosis but also in DVT [7, 8]. Tissue manipulation or dissection during operation can trigger local and systemic inflammation, which may be a causative mechanism of DVT [9]. Through promoting coagulation or inhibiting fibrinolysis, the inflammation cytokines may generate a hypercoagulable state and lead to thrombotic diseases [10]. Previous research has studied the association between DVT and multiple inflammation biomarkers in plasma, including C-reactive protein (CRP), high-sensitivity CRP, interleukin (IL)-1β, IL-6, IL-8, and tumor necrosis factor (TNF)-α [11–13].

The neutrophil to lymphocyte ratio (NLR) and the platelet to lymphocyte ratio (PLR) are biomarkers for systemic inflammation, which can be achieved easily through routine blood test without other expense. The predictive value of NLR and PLR in arterial and oncological diseases has been evaluated in various studies [14, 15]. However, the association of the plasma NLR or PLR level with the acute DVT after TJA is yet to be determined, which triggered us to identify the predictive or diagnostic value of NLR and PLR. We conducted this retrospective study to investigate the role of NLR and PLR in patients with acute DVT after TJA.

## Methods

### Study population

We included adult patients who underwent primary TKA or THA in Nanjing Drum Tower Hospital affiliated to Medical School of Nanjing University between March 2011 and March 2014. The diagnoses of the patients were hip or knee osteoarthritis, rheumatoid arthritis, fracture of femoral neck, avascular necrosis of femoral head, and so on. The patients with infectious or inflammatory diseases, hematological disorders, serious renal dysfunction, or hypohepatia as well as current use of immunosuppressive agents were excluded from this research. This retrospective study was approved by the Hospital Ethical Committee.

Surgeries were performed by three experienced surgeons. All the patients received 0.3 ml of low-molecular-weight heparin subcutaneously once daily until venography was performed. In addition, pressure pumps were used to squeeze bilateral lower limbs rhythmically as mechanical prophylaxis of DVT. All patients underwent regular rehabilitation program after surgery.

Venography was performed routinely at 3–5 days after the surgery by one doctor and reviewed by at least two experienced radiologists according to Robinov group’s criterion. Proximal DVT was defined as thrombosis at the level of popliteal vein or above. Thrombosis occurring within the calf veins was considered as distal DVT. Once DVT was confirmed, conventional thrombolysis treatment was performed. If no DVT was detected, patients would not receive any further anticoagulation treatment.

The blood specimen was collected from the peripheral venous before the operation and on the first morning after the operation. The blood routine test was performed in the clinical laboratory of Nanjing Drum Tower Hospital affiliated to Medical School of Nanjing University. Pre- and postoperative NLR and PLR were calculated according to the blood cell count. In addition, the perioperative D-dimer test and patients’ basic demographic and clinical characteristics (age, gender, body mass index (BMI), hypertension, diabetes mellitus, smoking, heart diseases, malignancy, and thrombosis history) were recorded.

### Statistical analysis

Statistical analysis was performed with the SPSS 22.0 software (SPSS Inc., Chicago, IL, USA). Continuous variables as mean ± standard deviation and categorical variables as numbers with percentage were shown. The Student *t* test and chi-square test were applied to compare the continuous variables and categorical variables respectively between different groups. The test of equal variance for continuous variables was performed before *t* test. Modified *P* values calculated by SPSS were adapted when equal variance was not assumed in some continuous variables (age, preoperative NLR/PLR, and postoperative platelet/PLR/D-dimmer). The independent association of NLR or PLR with DVT after adjustment for other variables was investigated by multiple logistic regression analyses. The odd ratio (OR) and 95% confidence intervals (CIs) were calculated for every associated variables. Receiver-operating characteristic (ROC) curve analysis was performed to identify the sensitivity and specificity of WBC, NLR, PLR, and D-dimer for the prediction of DVT. *P* < 0.05 was considered significant in all statistical analyses.

## Results

Finally, a total of 773 patients (290 TKA and 483 THA) were evaluated in this study. There were 224 males and 549 females. The patients averaged 64.1 ± 13.2 years of age (rang: 18-93 years). One hundred twenty patients (15.5%) were diagnosed with DVT by venography, including 19 patients with proximal DVT and 101 patients with distal DVT. Baseline demographic and clinical characteristics of the patients were summarized in Table 1. It was detected that advanced age (*P* < 0.001), BMI (*P* = 0.005), female gender (*P* = 0.002), and malignancy history (*P* = 0.013) were associated with DVT after TJA.

**Table 1:** Baseline demographic and clinical characteristics of patients

Subjec**t**s	With DVT *N* = 120	Without DVT *N* = 653	*P* value
Age (year)	68.8 ± 10.6	63.3 ± 13.5	< 0.001
Gender (female)	99 (82.5%)	450 (68.9%)	0.002
BMI	25.6 ± 4.2	24.4 ± 4.4	0.005
Hypertension	49 (40.8%)	225 (34.5%)	0.179
Insulin resistance	15 (12.5%)	70 (10.7%)	0.529
Smoking history	13 (10.8%)	58 (8.9%)	0.492
Heart disease	10 (8.3%)	43 (6.6%)	0.438
Malignancy	8 (6.7%)	14 (2.1%)	0.013
Thrombosis history	17 (14.2%)	65 (10.0%)	0.264

P<0.05 was considered statistically significant

Comparison about the perioperative laboratory examinations between the DVT and non-DVT groups is shown in Table 2. In patients with DVT, preoperative NLR (*P* = 0.030), postoperative NLR (*P* = 0.015), and the postoperative white blood cell count (WBC) (*P* < 0.001) were significantly higher in comparison to the non-DVT group. In contrast, postoperative PLR (*P* = 0.002) is significant lower in DVT group. No significant difference was detected for other variables between these two groups. When stratified by the extent of DVT (proximal and distal DVT), the level of postoperative NLR (*P* = 0.03) and WBC (*P* = 0.001) was significant higher in the proximal DVT group than that of the distal DVT group (Table 3).

**Table 2:** Perioperative laboratory data of patients

Subjects	With DVT *N* = 120	Without DVT *N* = 653	*P* value
Preoperative data			
HB (g/L)	125.7 ± 15.3	127.9 ± 14.5	0.127
RBC count (×10^12^/L)	4.1 ± 0.5	4.2 ± 0.5	0.052
WBC count (×10^9^/L)	6.4 ± 2.2	6.1 ± 1.9	0.133
Platelet count (×10^9^/L)	203.1 ± 62.7	198.1 ± 66.2	0.442
NLR	2.6 ± 2.3	2.3 ± 1.7	0.030
PLR	126.3 ± 69.6	117.2 ± 46.5	0.071
D-dimer (mg/L)	1.0 ± 1.8	0.9 ± 1.8	0.683
Postoperative data			
HB (g/L)	110.8 ± 14.1	108.6 ± 16.0	0.170
RBC count (×10^12^/L)	3.6 ± 0.5	3.6 ± 0.5	0.711
WBC count (×10^9^/L)	12.0 ± 2.6	10.9 ± 2.5	< 0.001
Platelet count (×10^9^/L)	158.8 ± 45.8	167.4 ± 59.9	0.132
NLR	9.5 ± 4.1	8.4 ± 4.9	0.015
PLR	131.2 ± 60.8	162.6 ± 110.0	0.002
D-dimer (mg/L)	2.5 ± 2.7	2.8 ± 5.4	0.427

*P* < 0.05 was considered statistically significant

**Table 3:** Distribution of WBC, NLR, and PLR between the subgroups of DVT

Subjects	Proximal DVT *N* = 19	Distal DVT *N* = 101	*P* value
Preoperative data			
WBC count (×10^9^/L)	6.5 ± 3.1	6.4 ± 2.0	0.885
NLR	3.01 ± 3.3	2.6 ± 2.1	0.405
PLR	133.6 ± 112.5	124.9 ± 58.9	0.621
Postoperative data			
WBC count (×10^9^/L)	13.6 ± 2.8	11.7 ± 2.4	0.003
NLR	12.4 ± 5.4	9 ± 3.6	0.001
PLR	138.2 ± 60.8	129.8 ± 57.0	0.584

*P* < 0.05 was considered statistically significant

**Fig. 1:**
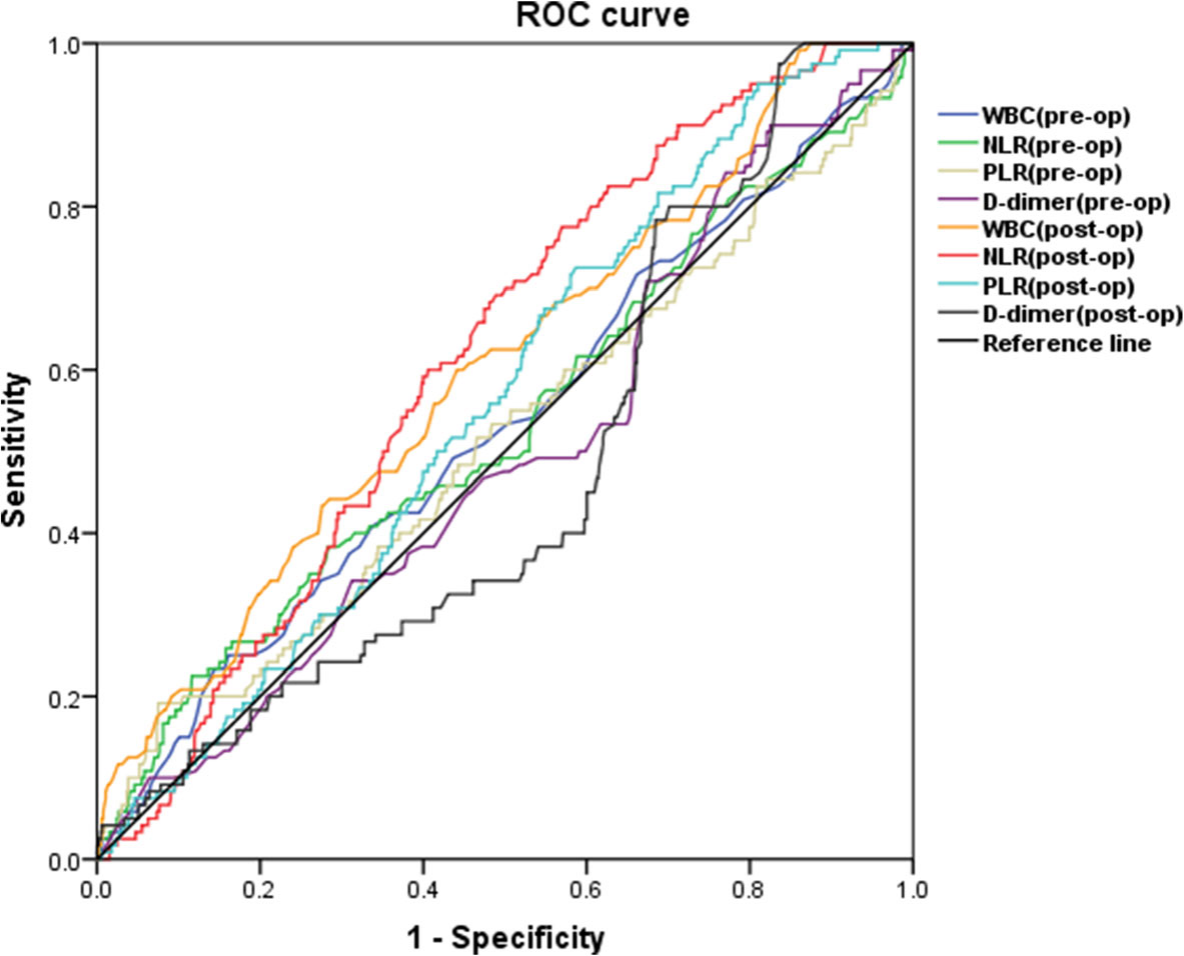
ROC curve of WBC, NLR, PLR, and D-dimer to predict DVT. The ROC curve analysis and AUC demonstrated the specificity and sensitivity of perioperative WBC, NLR, PLR, and D-dimer in predicting DVT were low

The results of multiple logistic regression analyses (Table 4) indicated that age (OR = 1.05, *P* < 0.005), gender (OR = 0.47, *P* = 0.005), BMI (OR = 1.06, *P* < 0.014), preoperative NLR (OR = 1.11, *P* < 0.035), postoperative NLR (OR = 1.20, *P* < 0.001), and PLR (OR = 0.99, *P* < 0.001) were independently associated with DVT. However, the ROC curve analysis and areas under the curve (AUC) demonstrated the specificity and sensitivity of perioperative WBC, NLR, PLR, and D-dimer in predicting DVT were low (Fig. 1 and Table 5).

**Table 4:** Multiple logistic regression analyses for predictors of DVT

Subjects	OR	95% CI	*P* value
Age	1.05	1.02–1.07	< 0.001
Gender	0.47	0.27–0.80	0.005
BMI	1.06	1.01–1.11	0.014
Malignancy	2.69	0.97–7.42	0.057
NLR (pre-op)	1.11	1.01–1.23	0.035
WBC (post-op)	1.04	0.95–1.15	0.416
NLR (post-op)	1.20	1.12–1.30	< 0.001
PLR (post-op)	0.99	0.98–0.99	< 0.001

*P* < 0.05 was considered statistically significant

**Table 5:** AUC of the ROC curve and 95% confidence interval of WBC, NLR, PLR, and D-dimer for DVT predicting

Subjects	AUC	95% CI	*P* value
WBC (pre-op)	0.531	0.473–0.589	0.275
NLR (pre-op)	0.533	0.473–0.592	0.256
PLR (pre-op)	0.513	0.453–0.573	0.647
D-dimer (pre-op)	0.495	0.440–0.551	0.871
WBC (post-op)	0.601	0.546–0.656	<0.001
NLR (post-op)	0.613	0.564–0.662	< 0.001
PLR (post-op)	0.561	0.510–0.611	0.035
D-dimer (post-op)	0.475	0.422–0.528	0.387

*P* < 0.05 was considered statistically significant

## Discussion

This is the first study aiming to identify the predictive value of NLR and PLR for acute DVT after primary TJA. We demonstrated that the high level of pre- and postoperative NLR and low level of postoperative PLR were significantly associated with DVT after TJA. However, the ROC curve analyses indicated NLR or PLR cannot predict TJA-induced DVT accurately.

Postsurgical inflammation response, occurring within hours of surgery, creates a prothrombotic environment. The underlying cellular and molecular mechanisms involve many kinds of cytokines such as CRP, IL-6, and TNF-α [9]. Studies with high level of evidence researching NLR as an inflammatory biomarker for predicting acute DVT after TJA is not yet available. Barker et al. reported a link between NLR and DVT after TKA with positive conclusion, but it is a pilot study with just 20 samples [16]. It has been shown in several studies that NLR was predictor of coronary artery diseases (CADs) and PE [14, 17]. In the present study, NLR before and after the operation were significantly greater in the patients with DVT. Multivariate logistic regression analysis indicated that NLR was an independent factor of DVT. In addition, the level of NLR in patients with proximal DVT was significantly higher compared with patients with distal DVT, which is consistent with the previous study [18]. The mechanism explaining the result is still unclear. It was proposed that NLR may be a better marker than WBC reflecting systemic inflammation after the operation. Increased cytokine release from activated neutrophils may be responsible for thrombus formation. Neutrophil extracellular traps (NETs) are released from stimulated neutrophils in a process known as NETosis, which has been observed in patients undergoing orthopedic surgeries [19]. NETs formation may play a critical role between inflammation and DVT [9, 20]. Further studies are required to evaluate the underlying mechanism between inflammation and DVT presence.

PLR initially served as a systemic inflammation biomarker to predict oncological diseases [15]. Recently, with the growing recognition that high platelet and low lymphocyte count are related to progression of atherosclerosis, which means PLR is a promising biomarker of CADs [21]. Although platelet activation and aggregation has attracted more interest in arterial thrombosis, increasing evidence indicated that platelets may also play an important role in DVT [22]. In our study, the level of preoperative PLR in the DVT group was higher, despite no significant difference. On the contrary, the level of postoperative PLR was significantly lower in the DVT group. The exact mechanism explaining the result is not clear. It is possible that there may be much more activation and consumption of the platelet in the DVT group due to the operation and DVT formation.

Despite the significant difference about NLR and PLR between two groups, the ROC curve analysis suggested the sensitivity and specificity of these two biomarkers for predicting acute DVT after TJA are limited. The level of NLR and PLR has significant individual difference among different patients and may be affected by many factors such as blood loss, transfusion, and drug use during the perioperative period.

Several limitations of our studies exist and deserve mention. Firstly, the present study was the retrospective design. Secondly, we only performed venography in the operated lower limbs, ignoring the possibility of DVT on the non-operated lower limb. DVT can also happen in the non-operated lower limbs although the incidence is rare. Thirdly, we did not measure other inflammatory biomarkers such as CRP and the correlation between NLR and other inflammatory biomarkers could not be assessed.

## Conclusion

In summary, the present study demonstrated significant association of perioperative NLR or PLR with acute DVT after TJA, but NLR or PLR cannot predict TJA-induced DVT accurately.

## Abbreviations

ACU: Areas under the curve
BMI: Body mass index
CADs: Coronary artery diseases
CIs: Confidence intervals
CRP: C-reactive protein
DVT: Deep vein thrombosis
HB: Hemoglobin
IL: Interleukin
NETs: Neutrophil extracellular traps
NLR: The neutrophil to lymphocyte ratio
OR: The odd ratio
PE: Pulmonary embolism
PLR: The platelet to lymphocyte ratio
Post-op: Postoperative
Pre-op: Preoperative
RBC: Red blood cell
ROC: Receiver-operating characteristic
THA: Total hip arhroplasty
TJA: Total joint arthroplasty
TKA: Total knee arthroplasty
TNF: Tumor necrosis factor

## References

[1] Won MH, Lee GW, Lee TJ, Moon KH (2011). Prevalence and risk factors of thromboembolism after joint arthroplasty without chemical thromboprophylaxis in an Asian population. J Arthroplast.

[2] Russell RD, Huo MH (2013). Apixaban and rivaroxaban decrease deep venous thrombosis but not other complications after total hip and total knee arthroplasty. J Arthroplast.

[3] Dua A, Desai SS, Lee CJ, Heller JA (2017). National trends in deep vein thrombosis following total knee and total hip replacement in the United States. Ann Vasc Surg.

[4] Hou H, Ge Z, Ying P, Dai J, Shi D, Xu Z, Chen D, Jiang Q (2012). Biomarkers of deep venous thrombosis. J Thromb Thrombolysis.

[5] Shi D, Xu X, Xu Z, Nakamura T, Pang Y, Yao C, Wang F, Chen D, Dai J, Jiang Q (2014). P-selectin: an unpredicted factor for deep vein thrombosis after total hip arthroplasty. Biomed Res Int.

[6] Xu Z, Shi D, Zhang C, Chen D, Dai J, Teng H, Jiang Q (2013). Postoperative plasma D-dimer value for predicting deep venous thrombosis following hip arthroplasty with nadroparin prophylaxis. Hip Int.

[7] Reganon E, Vila V, Martinez-Sales V, Vaya A, Mira Y, Ferrando F, Aznar J (2007). Sialic acid is an inflammation marker associated with a history of deep vein thrombosis. Thromb Res.

[8] Matos MF, Lourenco DM, Orikaza CM, Bajerl JA, Noguti MA, Morelli VM (2011). The role of IL-6, IL-8 and MCP-1 and their promoter polymorphisms IL-6 -174GC, IL-8 -251AT and MCP-1 -2518AG in the risk of venous thromboembolism: a case-control study. Thromb Res.

[9] Albayati MA, Grover SP, Saha P, Lwaleed BA, Modarai B, Smith A (2015). Postsurgical inflammation as a causative mechanism of venous thromboembolism. Semin Thromb Hemost.

[10] Osnes LT, Westvik AB, Joo GB, Okkenhaug C, Kierulf P (1996). Inhibition of IL-1 induced tissue factor (TF) synthesis and procoagulant activity (PCA) in purified human monocytes by IL-4, IL-10 and IL-13. Cytokine.

[11] Zacho J, Tybjaerg-Hansen A, Nordestgaard BG (2010). C-reactive protein and risk of venous thromboembolism in the general population. Arterioscler Thromb Vasc Biol.

[12] Christiansen SC, Naess IA, Cannegieter SC, Hammerstrom J, Rosendaal FR, Reitsma PH (2006). Inflammatory cytokines as risk factors for a first venous thrombosis: a prospective population-based study. PLoS Med.

[13] Reitsma PH, Rosendaal FR (2004). Activation of innate immunity in patients with venous thrombosis: the Leiden thrombophilia study. J Thromb Haemost.

[14] Papa A, Emdin M, Passino C, Michelassi C, Battaglia D, Cocci F (2008). Predictive value of elevated neutrophil-lymphocyte ratio on cardiac mortality in patients with stable coronary artery disease. Clin Chim Acta.

[15] Gu X, Sun S, Gao XS, Xiong W, Qin S, Qi X, Ma M, Li X, Zhou D, Wang W (2016). Prognostic value of platelet to lymphocyte ratio in non-small cell lung cancer: evidence from 3,430 patients. Sci Rep.

[16] Barker T, Rogers VE, Henriksen VT, Brown KB, Trawick RH, Momberger NG, Lynn RG (2016). Is there a link between the neutrophil-to-lymphocyte ratio and venous thromboembolic events after knee arthroplasty? A pilot study. J Orthop Traumatol.

[17] Kayrak M, Erdogan HI, Solak Y, Akilli H, Gul EE, Yildirim O, Erer M, Akilli NB, Bekci TT, Aribas A (2014). Prognostic value of neutrophil to lymphocyte ratio in patients with acute pulmonary embolism: a restrospective study. Heart Lung Circ.

[18] Bakirci EM, Topcu S, Kalkan K, Tanboga IH, Borekci A, Sevimli S, Acikel M (2015). The role of the nonspecific inflammatory markers in determining the anatomic extent of venous thromboembolism. Clin Appl Thromb Hemost.

[19] McIlroy DJ, Jarnicki AG, Au GG, Lott N, Smith DW, Hansbro PM, Balogh ZJ (2014). Mitochondrial DNA neutrophil extracellular traps are formed after trauma and subsequent surgery. J Crit Care.

[20] Kimball AS, Obi AT, Diaz JA, Henke PK (2016). The emerging role of NETs in venous thrombosis and immunothrombosis. Front Immunol.

[21] Li H, Zhou Y, Ma Y, Han S, Zhou L (2017). The prognostic value of the platelet-to-lymphocyte ratio in acute coronary syndrome: a systematic review and meta-analysis. Kardiol Pol.

[22] Montoro-Garcia S, Schindewolf M, Stanford S, Larsen OH, Thiele T (2016). The role of platelets in venous thromboembolism. Semin Thromb Hemost.

